# Investigating potato flour processing methods and ratios for noodle production

**DOI:** 10.1002/fsn3.4058

**Published:** 2024-03-05

**Authors:** Ariel Buzera, Evelyne Gikundi, Napoleon Kajunju, Jackson Ishara, Irene Orina, Daniel Sila

**Affiliations:** ^1^ Department of Food Science & Technology Université Evangelique en Afrique (UEA) Bukavu Sud‐Kivu Democratic Republic of the Congo; ^2^ Department of Food Science & Technology Jomo Kenyatta University of Agriculture and Technology (JKUAT) Nairobi Kenya; ^3^ Department of Food Science and Technology Makerere University Kampala Uganda

**Keywords:** cooking properties, noodles, potato flour, processing methods, sensory analysis, texture properties

## Abstract

A partial substitution of wheat flour with potato flour processed by various procedures was used to determine an optimal potato pretreatment method for noodle processing. Wheat flour was substituted with 10%, 30%, and 50% potato flour. Potato flour (PF) was processed using two different methods, including freeze‐drying (FD) and low‐temperature blanching, then oven drying (LTB_OD). The results showed that substituting wheat flour with freeze‐dried (FD) flour (44.29 μm) significantly decreased the mean particle size of the blended flour, while LTB_OD flour (223.09 μm) increased the mean particle size. The pasting properties of wheat flour significantly improved when potato flour was added, with FD flour blends having the highest results. The highest dough development time (14.46 min) was attained when LTB_OD potato flour was substituted up to 50%. The microstructure images showed a poor and discontinuous gluten framework when potato flour content reached 50%. Adding potato flour decreased noodles' brightness (L*) while increasing their yellowness (b*). Noodles made from wheat and LTB_OD flour blends resulted in the highest cooking loss. The texture properties of noodles deteriorated when potato flour content reached 30%. Substituting up to 30% with freeze‐dried flour and 10% LTB_OD resulted in noodles with the highest overall liking scores. The study suggests that for optimal noodle processing, substituting wheat flour with FD potato flour is more favorable than using LTB_OD, as it improves particle size, pasting properties, and overall liking scores while minimizing adverse effects on texture and cooking loss.

## Graphical abstract

This study investigated the effects of substituting wheat flour with potato flour produced using different processing methods. The results revealed that replacing wheat flour with freeze‐dried potato flour improved the blended flour's particle size, pasting properties, and overall liking scores for noodles. In contrast, using low‐temperature blanching and oven‐dried potato flour led to adverse effects on texture and cooking loss, suggesting that freeze‐dried potato flour is a more favorable option for optimizing noodle processing.

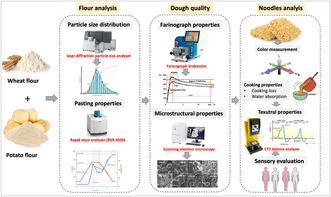

## 1 INTRODUCTION

Potatoes (*Solanum tuberosum* L.) are a major food crop consumed by over a billion people worldwide (Devaux et al., 2021). The Food and Agriculture Organization (FAO) has highly recommended potatoes as a food security crop to fight uncertain food supplies, growing populations, and increasing demand for food (FAO, 2008). Potatoes are inherently perishable. Substantial losses occur during harvesting, storage, distribution, and marketing (Degebasa, 2020). To mitigate postharvest losses of potato tubers and increase their use in food production, they can be processed into flour (Lingling et al., 2019).

To prepare potato flour, slices of peeled potatoes and mashed potatoes are dried in hot air or drum dryers, then ground and sieved (Yadav et al., 2006). Aside from carbohydrates, potato flour is a rich source of vitamins, minerals, and amino acids (Ju et al., 2017). Additionally, potatoes contain polyphenols and flavonoids, which benefit human health (Ezekiel et al., 2013). In addition to being a thickener, potato flour enhances food color and flavor. It is incorporated in sauces, fabricated snacks, bakery products, gravy, extruded products, and soup mixes (Yadav et al., 2006). In wheat‐based products, potato flour has been used to reduce the dependence on wheat flour and reduce the allergenicity of wheat gluten (Meng et al., 2022).

Noodles are wheat‐based products that can be enriched with potato flour (Nawaz et al., 2019) and (Pu et al., 2017). Noodles are convenient, easy to cook, delicious, low in cost, and maintain their quality for an extended period, which suits the busy lifestyle of students and the working population (Onyema et al., 2014; Sikander et al., 2017). Noodles are traditionally made by mixing wheat flour with water, then sheeting and cutting into strips (Fu, 2008). Gluten protein and wheat starch are essential to noodles' quality. Gluten acts as the skeleton, while wheat starch is the noodle dough's filler. Gluten controls dough strength and elasticity (Kovacs et al., 2004). Gluten‐free potato flour will impact the quality of noodles in terms of protein quality, quantity, and starch quality (degree of gelatinization). Studies have shown that methods used for potato production determine the physicochemical properties of the resulting flour (Bao et al., 2021; Buzera et al., 2022). However, limited information is available on the properties of noodles made from potato flour processed in different ways. This study examined the effects of partially substituting potato flour processed in different ways with wheat flour on noodle cooking, textural and sensory qualities.

## 2 MATERIALS AND METHODS

### 2.1 Samples' collection

Wheat flour (Pembe) was purchased from a local supermarket in Nairobi, Kenya. The Shangi potato variety was procured from a farmer in Nyandarua County, Kenya.

### 2.2 Potato flour preparation

**FIGURE 1:**
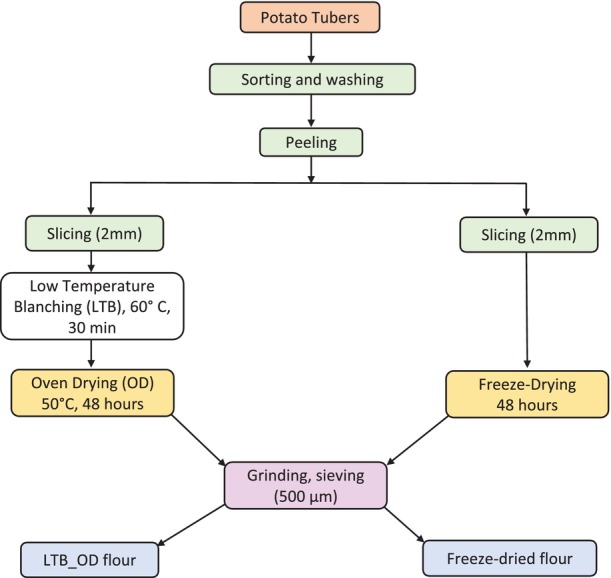
Overview chart of potato flour preparation.

The potato tubers were cleaned, peeled, and thinly sliced into 2‐mm thick slices. One batch of sliced potatoes was freeze‐dried (FD) (Lyovapor L‐200 Pro, BUCHI, Flawil, Switzerland) for 48 h. The initial temperature during freeze‐drying (FD) was—41°C and 0.11 millibars, while the final temperature was—47°C and 0.055 millibars. Another batch of potato slices was blanched at 60°C for half an hour before being oven‐dried at 50°C for 48 h (LTB_OD). The dried slices were ground into flour using a blender (Vitamix 5200 Blender, Professional‐Grade, Cleveland, OH, USA) and sieved through a 500‐μm sieve. A polyethylene ziplock bag was used to store the flours until they could be analyzed. The process flowchart of the potato flour preparation is shown in Figure 1.

### 2.3 Formulation of composite flour

A mixture of wheat flour and potato flour was prepared in different percentages, as detailed in Table 1. Each of the two types of potato flour, freeze‐dried (FD) and LTB_OD potato flour, substituted wheat at 10%, 30%, and 50% ratios. The control sample was made of 100% wheat flour.

**TABLE 1:** Wheat and potato flours' blend mixing ratios (%).

Sample	Mixing ratio
Wheat: Potato	10:0 (control wheat)
Wheat: 10% Potato	9:1 (10% FD, 10% LTB_OD)
Wheat: 30% Potato	7:3 (30% FD, 30% LTB_OD)
Wheat: 50% Potato	5:5 (50% FD, 50% LTB_OD)

Abbreviations: FD, freeze‐dried flour; LTB_OD, low‐temperature blanched followed by oven drying.

### 2.4 Composite flours' analysis

#### 2.4.1 Particle size distribution

The particle size distribution of the flour was determined, as described by Stachowiak et al. (2021). A SALD‐2300 laser diffraction measurement system (Shimadzu, Kyoto, Japan) with a cyclone injection unit was used. This device measures particle sizes between 17 nanometers (nm) and 2500 micrometers (μm). A hopper containing approximately 2 g of potato flour was connected to a laser beam using an ejector tool to draw particles across the laser beam. The scattered light intensity distribution pattern generated by a laser‐irradiated sample determines the particle size distribution. A WingSALD II analysis software was used to analyze the results (version 3.1.0, Shimadzu, Kyoto, Japan).

#### 2.4.2 Pasting properties

The pasting properties of potato flour blends were estimated using a Rapid Visco‐Analyzer (RVA 4500) (Newport Scientific Pty. Ltd., Warriewood, Australia) according to the method described by Gelencsér (2009). The parameters measured were peak viscosity, trough viscosity, breakdown viscosity, final viscosity, setback viscosity, and final viscosity (Balet et al., 2019).

#### 2.4.3 Farinograph properties

The properties of doughs prepared from a mixture of wheat flour and potato flour were determined by Brabender farinograph model 820603 (Brabender OHG, Duisberg, Germany). The thermostat was maintained at 30°C, and the commercial flour with 15% native starch was mixed in a 300‐g mixing bowl without water for 5 min in case of homogeneous materials. Following that, 63 revolutions per minute (rpm) of the mixing speed was set for 30 min, and water was poured into the right‐front corner of the bowl from a large burette. Dough development time, stability time, water absorption, and softening degree of the dough were determined (Zhang et al., 2022).

#### 2.4.4 Microstructural properties of the dough

The dough microstructure images were captured using a benchtop scanning electron microscope (model JCM‐7000 NeoScope Benchtop, JEOL Ltd, Tokyo, Japan). The inner part of the dough was collected and placed on an aluminium stub using double‐sided adhesive tape. A magnification of 270× and an accelerating potential of 15 kV were applied during the image capture.

### 2.5 Noodle production

The noodles were processed using a method previously described by Tiony and Irene (2021) with some modifications. One kilogram (1 kg) of each flour blend (Table 1) was mixed with 360 mL water, 16 g table salt, 2 g sodium bicarbonate, and 2 g sodium tripolyphosphate in a rotary mixer (JHMZ‐200, East Fude Technology Development Center, Beijing, China). The dough was formed into a thick sheet and allowed to rest in a plastic bag for 20 min. The dough was then reduced in thickness by passing through an extension roller. A shredder (JMTD 168/140, East Fude Technology Development Center, Beijing, China) was used to shred the thin dough sheet. After the shredding of the dough, pieces of 10 cm were cut out and steamed for 20 min before being deep‐fried for 30 s at 140°C. A sealed airtight bag was used to pack the instant fried noodles once they had been cooled at room temperature.

### 2.6 Color determination of the noodles

A HunterLab's EZ spectrophotometer, ColorFLex, USA, was used to measure the noodles' color (Wafula et al., 2021). First, white and black tiles were used for instrument calibration. The strands of noodles were placed tightly inside a transparent glass cup topped with an opaque cover, which acted as a light trap. After the noodles were packed, the clear glass was placed on the port. The intensity of color was measured using a spectrophotometer by flashing a light onto the sample. Three‐dimensional (3D) color values were reported on the following rating scale: Lightness (L*), redness (a*), and yellowness (b*). The measurements were undertaken in triplicate.

### 2.7 Cooking properties of noodles

#### 2.7.1 Water absorption capacity (WA)

The weight of cooked noodles was evaluated using a method described by Yadav et al. (2014) with slight modifications. Approximately three grams (3 g) of noodles was boiled for 4 min in 200 mL of water until cooked (until they became transparent in the center). After cooking, the noodles were rinsed with distilled water, drained for five minutes, and weighed immediately. The following equation calculated the water absorption:

(1) $$\text{Water}\;\text{absorption}=\frac{\left(W_{1}-W_{0}\right)}{W_{0}}\times 100$$

where *W*_0_ is the mass of the noodles before cooking (g) and *W*_1_ is the mass after cooking (g).

#### 2.7.2 Cooking loss

The cooking loss was estimated according to the method described by Yang et al. (2022). Ten grams (10 g) of noodles was boiled in 500 mL of deionized water until cooked. The remaining cooking water was collected into a volumetric flask (500 mL) and topped to volume with distilled water. Then, 50 mL of the solution was placed in a 250‐mL beaker (predried to constant weight before use). After that, the beaker was placed in a hot oven dryer at 105°C until it achieved a constant weight. The following equation was used to calculate the cooking loss:

(2) $$\text{Cooking}\;\text{loss}=10\times \frac{\left(W_{4}-W_{3}\right)}{W_{2}}\times 100$$

where *W*_2_ is the mass of the noodles before cooking (g), *W*_3_ is the mass of the beaker before drying (g), and *W*_4_ is the mass of the beaker after oven drying with dried substances of the cooking water (g).

### 2.8 Textural properties of cooked noodles

Textural properties of cooked noodles were evaluated using a texture analyzer (CT3, Brookfield, USA), according to Thuy et al. (2020). A wedge‐shaped probe (part number TA‐ PFS‐C) was used for texture profile analysis (TPA). The noodles were cooked in boiling deionized water (approximately 4 min), followed by cooling for 1 min under running distilled water and draining off the water before measurement. A random selection of five strands of cooked noodles was placed on the fixture base table parallel to the long edge of the probe. A force–time curve of the thermal profile analysis (TPA) was used to determine the hardness, resilience, springiness, cohesiveness, gumminess, and chewiness. Hardness refers to the maximum force recorded during the first compression cycle. Resilience is measured after the first penetration is withdrawn before the waiting period is started. The springiness of a sample is determined by how long it takes the sample to recover its height between the bites. Cohesiveness is calculated by dividing the positive area of the second cycle by the first cycle (Funami & Nakauma, 2022).

### 2.9 Sensory evaluation of cooked noodles

Sensory evaluation of cooked noodles was conducted based on the method proposed by Ibitoye et al. (2013) with slight modifications. All noodles' samples were cooked and served to 40 untrained panellists. All samples were randomly ordered and coded with random numbers. A 9‐point hedonic rating scale was used to determine preference for noodle samples, with 9 being rated as “like extremely” and 1 being “dislike extremely” for appearance, texture, aroma, taste, and general acceptability.

### 2.10 Statistical analysis

The results of all experiments were reported as the mean ± standard error. Analysis of variance (ANOVA) was performed using SPSS 18.0 (SPSS Inc., Chicago, USA), and Tukey's test at a significance level of .05 was conducted to separate the means.

## 3 RESULTS AND DISCUSSION

### 3.1 Particle size distribution of composite flours

**FIGURE 2:**
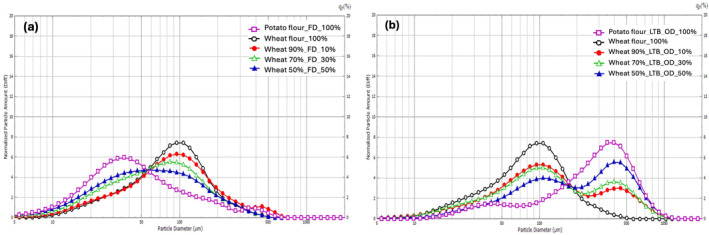
Particle size distribution curves of wheat–potato flour blends. (a) Freeze‐dried flour (FD). (b) Low‐temperature blanching then oven‐drying flour (LTB_OD).

Particle size is a crucial factor affecting flour product quality and functionality (Tian et al., 2022). The particle size distribution of the various composite flours is shown in Figure 2 and Table 2. Freeze‐dried potato flour and wheat flour displayed unimodal distribution curves with mean particle size diameters of 44.29 ± 0.41 and 85.72 ± 0.70 μm, respectively (Figure 2a). Freeze‐dried potato flour had a smaller particle size than wheat flour. A significant decrease in mean particle size was observed when wheat flour was substituted with freeze‐dried potato flour, while LTB_OD flour increased the mean particle of the composite flours, as seen in Table 2. Ahmed et al. (2014) observed that flours with a homogeneous particle size distribution could produce foods with well‐defined functional properties. The LTB_OD flour displayed a bimodal distribution with a mean particle size of 223.09 ± 0.39 μm (Figure 2b). The larger particle size observed in LTB_OD flour compared to freeze‐dried flour is attributed to the blanching (at 60°C for 30 min) of potato tubers during the flour processing. During the heat treatment, starch granules absorb water and swell, resulting in larger particles (Kim & Qin, 2014).

**TABLE 2:** Particle size distribution of composite wheat and potato flour.

Samples	*D10* (μm)	*D50* (μm)	*D90* (μm)	Mean (μm)
**Wheat flour 100%**	32.84 ± 0.69^c^	91.34 ± 0.50^de^	195.68 ± 0.69^cd^	85.72 ± 0.70^de^
**FD_Potato flour 100%**	14.80 ± 0.24^e^	41.11 ± 0.04^h^	162.53 ± 1.22^d^	44.29 ± 0.41^g^
Wheat 90%_FD_Potato 10%	24.94 ± 0.89^d^	85.48 ± 1.45^ef^	226.88 ± 1.58^c^	79.48 ± 1.36^ef^
Wheat 70%_FD_Potato 30%	18.57 ± 0.46^e^	67.25 ± 1.34^fg^	187.32 ± 5.92^d^	60.72 ± 1.43^fg^
Wheat 50%_FD_Potato 50%	15.83 ± 0.12^e^	55.85 ± 0.45^gh^	193.52 ± 17.48^cd^	54.18 ± 1.50^g^
**LTB_OD Potato flour 100%**	50.08 ± 0.01^a^	281.24 ± 1.21^a^	576.57 ± 2.03^a^	223.09 ± 0.39^a^
Wheat 90%_LTB_OD Potato 10%	28.64 ± 0.02^cd^	107.11 ± 1.42^cd^	464.02 ± 1.34^b^	108.79 ± 0.52^d^
Wheat 70%_LTB_OD Potato 30%	28.40 ± 0.63^cd^	115.49 ± 1.01^c^	495.19 ± 9.87^b^	120.05 ± 0.93^c^
Wheat 50%_LTB_OD Potato 50%	37.96 ± 2.61^b^	165.89 ± 11.93^b^	544.18 ± 9.11^a^	155.57 ± 7.37^b^

*Note*: The results are the means of three determinations ± standard error. A mean value with a different letter in the same column indicates a significant difference (*p* < .05, *n* = 3).

The bold parts represent the 100% values for Wheat flour, Freeze‐dried flour and LTB_OD flour, respectively.

Abbreviations: FD, Freeze‐dried; LTB_OD, low‐temperature blanching then oven drying.

### 3.2 Pasting properties of composite flours

**FIGURE 3:**
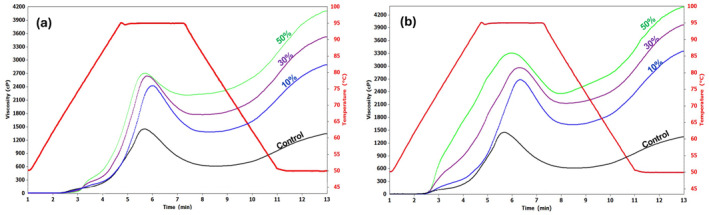
Pasting profile curves of wheat flour and the different blends. (a) Low‐temperature blanching (60°C) potato flour, (b) Freeze‐dried potato flour, and Control‐100% wheat.

Pasting properties refer to how flour behaves when heated in water. It affects the flour's texture, digestibility, and functionality (Ocheme et al., 2018). This study determined the pasting profiles of wheat–potato composite flours to evaluate their suitability for noodles. Wheat–potato flour blends are illustrated in Figure 3 and Table 3. Except for the breakdown viscosity, the composite flours had significantly higher pasting parameters than the control (100% wheat flour). A higher peak viscosity, trough viscosity, final viscosity, and setback viscosity were also observed when potato flour was added to wheat flour. The viscosity of potato starch is higher than that of starch from cereal grains, such as wheat, rice, and corn (Waterschoot et al., 2015). Amylopectin is bound to phosphate groups in potato starch, resulting in the starch's high swelling power (Waterschoot et al., 2015). As phosphate molecules repel one another, hydration increases as the extent of bonding in the crystalline structure is weakened (Karim et al., 2007; Noda et al., 2004).

**TABLE 3:** Pasting properties of wheat and potato flour blends.

Samples	Peak viscosity (cP)	Trough viscosity (cP)	Breakdown viscosity (cP)	Final viscosity (cP)	Setback viscosity (cP)	Pasting temperature (°C)
Wheat flour (100%)	1450.7 ± 53.99^e^	610.0 ± 17.04^f^	840.67 ± 37.35^c^	1356.0 ± 32.14^f^	746.0 ± 26.23^e^	86.12 ± 0.28^a^
Wheat 90%_FD_Potato 10%	2595.3 ± 53.44^c^	1562.3 ± 44.86^d^	1033.0 ± 12.12^a^	3257.0 ± 60.66^d^	1694.7 ± 27.39^c^	70.98 ± 0.02^c^
Wheat 70%_FD_Potato 30%	2936.0 ± 21.50^b^	2096.0 ± 48.49^b^	840.0 ± 29.36^c^	3903.3 ± 42.60^b^	1807.3 ± 20.95^b^	69.86 ± 0.23^d^
Wheat 50%_FD_Potato 50%	3294.3 ± 27.70^a^	2327.7 ± 31.84^a^	966.6 ± 9.35^ab^	4342.0 ± 52.63^a^	2014.3 ± 21.06^a^	69.32 ± 0.04^d^
Wheat 90%_LTB_Potato 10%	2354.7 ± 34.91^d^	1342.7 ± 24.73^e^	1012.0 ± 10.26^a^	2865.0 ± 43.06^e^	1522.3 ± 18.34^d^	86.67 ± 0.46^a^
Wheat 70%_LTB_Potato 30%	2626.0 ± 21.59^c^	1761.0 ± 11.53^c^	865.0 ± 10.59^bc^	3503.0 ± 15.17^c^	1742.0 ± 4.16^bc^	75.88 ± 0.02^b^
Wheat 50%_LTB_Potato 50%	2764.3 ± 51.16^bc^	2153.0 ± 70.69^ab^	611.3 ± 27.72^d^	4156.3 ± 61.93^a^	2003.3 ± 13.48^a^	75.07 ± 0.02^b^

*Note*: The results are the means of three determinations ± standard error. A mean value with a different letter in the same column indicates a significant difference (*p* < .05, *n* = 3).

Abbreviations: FD, Freeze‐dried; LTB_OD, Low‐temperature blanching then oven drying.

### 3.3 Farinograph properties

Farinograph properties of wheat flour and potato flour blend at 10%, 30%, and 50% are shown in Table 4. Adding potato flour to wheat flour significantly changed the dough mixing behavior. The results showed that water absorption gradually increased when the quantity of potato flour blends increased. Water absorption (WA) is the amount of water required to develop dough to a certain consistency (500 Brabender units (BU)) when the gluten is fully developed (Fu et al., 2017). LTB_OD potato flour blend absorbed more water than the FD potato flour blend. This could probably be due to the pre‐gelatinized starch of the LTB_OD flour. Whitney and Simsek (2020) reported that pre‐gelatinized starch generated during the production of potato flour increases water absorption. Xu et al. (2022) reported that a partially gelatinized starch (starch heated at 60°C) might have a greater ability to bind water molecules due to exposed hydrophilic groups. Therefore, the LTB_OD potato flour, probably with a high degree of starch gelatinization compared to freeze‐dried potato flour, could be responsible for increased water absorption of composition. The increase in water absorption values in composite flour may also be related to higher crude fiber in potato flour, which has hydrophilic components (Hasmadi et al., 2020). Goldstein et al. (2010) mentioned that gelatinization might cause an increase in cellulose fiber, resulting in a high water absorption of the dough.

**TABLE 4:** Dough quality of wheat/potato flour blends determined by the farinograph.

Treatment	Water absorption (%)	Development time (min)	Stability (min)	Softening degree (FU)
Control (100% wheat)	63.20	1.73	7.82	58.00
Wheat 90%_FD_Potato 10%	63.00	2.02	5.55	49.00
Wheat 70%_FD_Potato 30%	63.60	7.08	9.70	120.00
Wheat 50%_FD_Potato 50%	63.70	7.08	9.73	97.00
Wheat 90%_LTB_Potato 10%	64.10	1.76	2.63	80.00
Wheat 70%_LTB_Potato 30%	64.50	8.20	10.55	84.00
Wheat 50%_LTB_Potato 50%	67.50	14.46	11.65	50.00

Abbreviation: FQN, Farinogram quality number.

Dough development time increased significantly when the potato flour increased from 10% to 50%. Dough development time is when the dough is formed and achieves the consistency of 500 BU. The time that gluten is formed and the normal consistency of dough reaches 500 BU (Sun et al., 2022). The highest dough development time was attained when LTB_OD potato flour was substituted up to 50%. This could be due to the heterogeneity (bimodal distribution) of particle size of LTB_OD flour previously reported in the study. Flour with a bimodal distribution, with probably different particle sizes, would hydrate progressively, resulting in inconsistent water distribution in the dough (Sarker et al., 2008). To reach maximum dough consistency, all flour particles must be hydrated, which takes longer when particles are not homogeneous, resulting in a longer mixing time. The longer mixing time weakens the system and the breaking down of the dough (Sarker et al., 2008). The above finding can be supported by the scanning electron microscopy (SEM) images in the next Section 3.4.

Dough stability is when dough keeps its consistency with the continuous mixing process. The higher the values, the stronger the dough. Our results showed that adding potato flour at 10% reduced the stability of the dough, which indicates that the gluten strength of the blends was weakened. On the other hand, the values tended to increase rapidly from 30% for all the types of potato flour, and the highest value was observed in the LTB_OD potato flour blend at 50%. This suggests that dough containing LTB_OD caused decreased elasticity. Miyazaki and Morita (2005) reported that dough containing a larger amount of potato starch caused a decrease in elasticity. This result explains that LTB_OD also does not bind readily with gluten to form elastic dough. Sarker et al. (2008) studied dough characteristics of mixtures of wheat flour and potato starch prepared from three cultivars and found that dough stability significantly increased with an increase in potato starch. The same observation has been recorded with wheat–taro composite flour (Ammar et al., 2009).

The dough softening degree increased significantly with the potato flour addition. Liu et al. (2016) reported that the degree of softening increased with the addition of potato flour to mixed flour. Due to the gluten‐free nature of potato flour, it cannot form a strong gluten network, resulting in a dough that is not as elastic and cohesive as one made with wheat flour. Li et al. (2020) reported the same with the Chinese yam. Verwimp et al. (2006) reported that gluten's ability to recombine and its strength were weakened, resulting in shortened dough stability time, increased softening degree of blends, and decreased four quality coefficients.

### 3.4 Microstructure images of the dough substituted with different levels of potato flour

**FIGURE 4:**
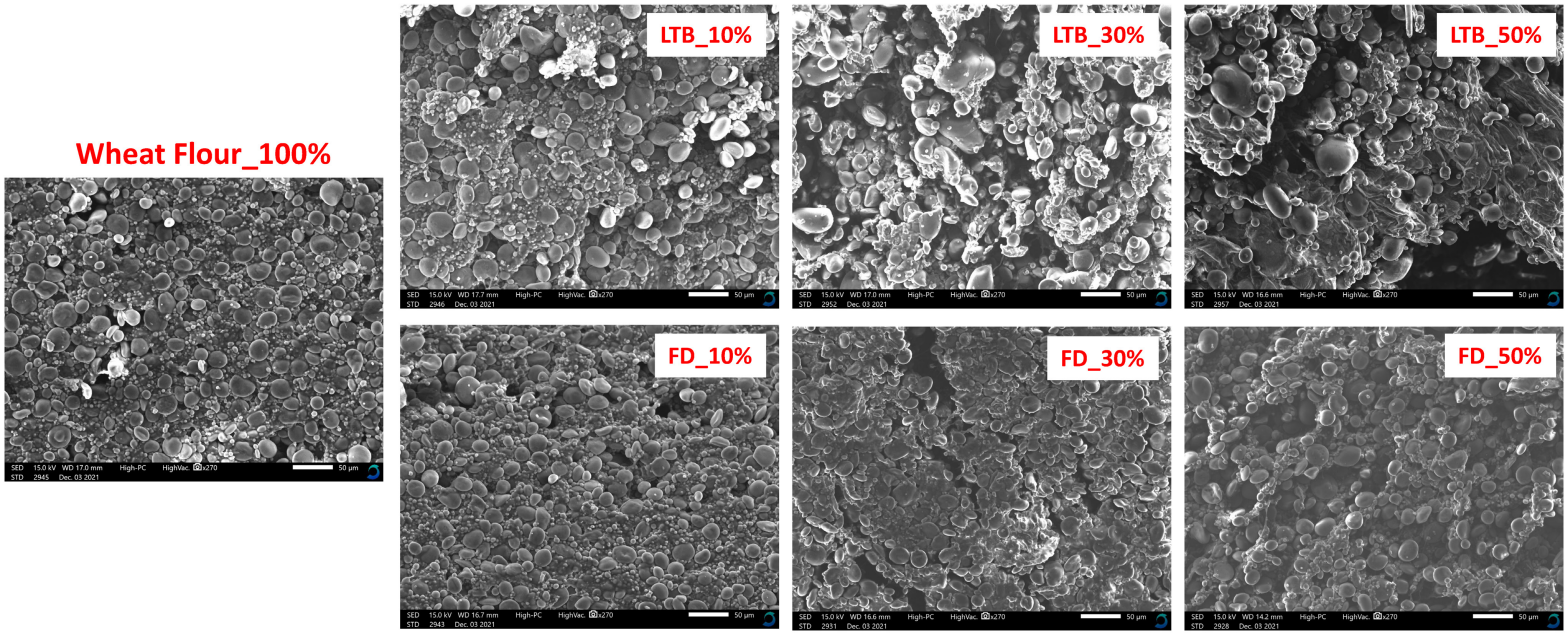
Scanning electron micrographs of dough substituted with different levels of potato flour (270×).

The scanning electron micrographs of the dough samples are shown in Figure 4. The control sample (wheat dough 100%) was characterized by small and large starch granules tightly dispersed in a network structure. Similar observations of wheat dough have been previously reported (Li et al., 2020; Xu et al., 2022). Xu et al. (2017) reported gluten networks in wheat dough are more continuous and exhibit distinct gluten film structures that enclose starch particles. The wheat dough sample showed a compact microstructure with fewer hollows and voids. With the addition of potato flour from 10% to 50%, the continuity of the gluten network structure becomes fragile, and the ability to wrap the starch granules becomes lower. When the added amount of LTB_OD potato flour was 10%, it could be seen that starch granules started separating. The effect was more noticeable when the amount of LTB_OD potato flour was 30% and 50%; more hollows and porous structures were visible. This can be associated with the mean particle size of LTB_OD potato flour, which was way larger than that of freeze‐dried potato flour. These findings agree with Xu et al. (2022), who reported that more membrane‐like structures were observed in partially gelatinized potato starch granules dough than in native‐gluten model dough. Zou et al. (2020) explained this by stating that gelatinizing starch particles could form a three‐dimensional (3D) network. Substituting FD potato flour with wheat flour resulted in fewer hollows and a less porous structure. The SEM results are consistent with the pasting properties discussed earlier. Cao et al. (2020) reported that due to the substitution of potato flour, the dough had different viscoelasticity due to the breakdown of the gluten network.

### 3.5 Noodles' properties

#### 3.5.1 Color properties of noodles

**FIGURE 5:**
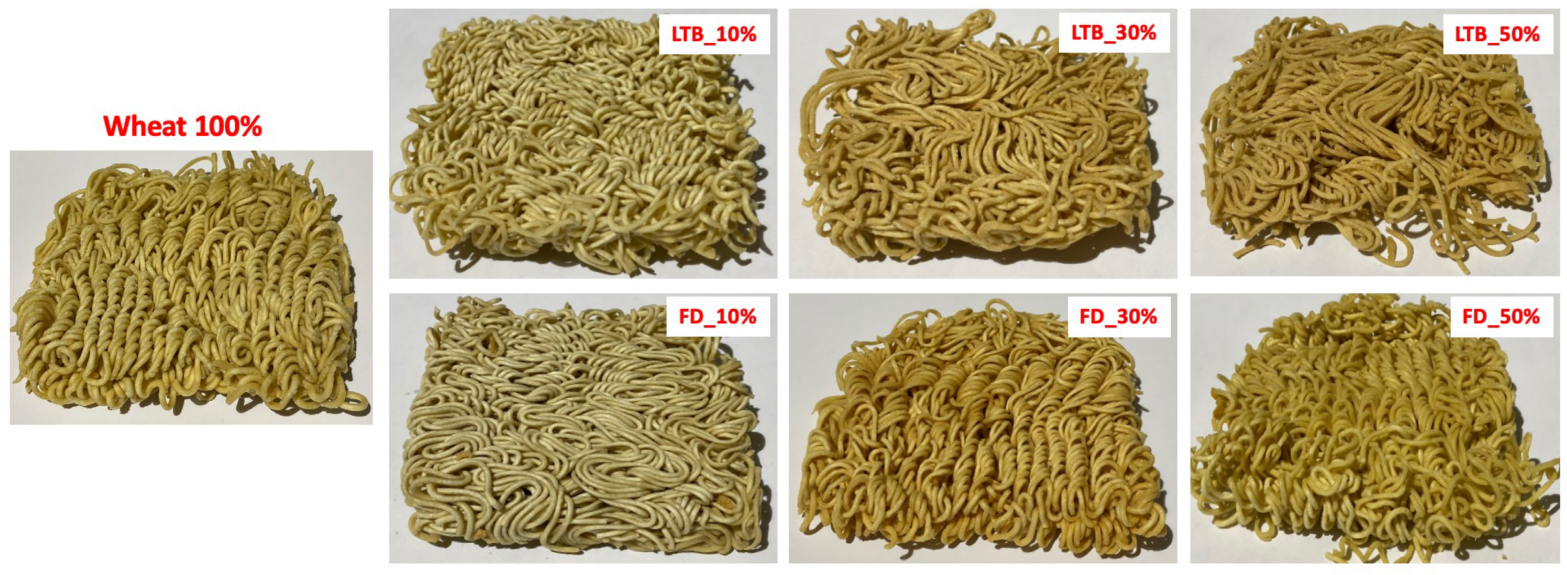
Image of noodles with different levels of different processed potato flours. FD, Freeze dried; LTB, Low‐temperature blanching then oven drying.

The color of noodles is an important quality determinant. Consumers are first attracted to bright and uniform‐colored noodles without faded patches (Li et al., 2017). The results of color measurement are shown in Table 5 and Figure 5. Control with 100% wheat flour had the highest brightness (67.77 ± 0.23), while wheat flour substituted with 50% LTB_OD potato flour had the lowest brightness (56.49 ± 0.01). There were significant decreases in noodle brightness (L*) with the addition of potato flour. Among the flour blends, freeze‐dried flour gave brighter noodles than LTB‐OD flour noodles. Yang (2020) reported that ingredients other than wheat flour decrease the L* value of noodles. These results agree with Desai et al. (2018) and Kowalczewski et al. (2015), who reported a decrease in noodles' brightness after incorporating fish powder and potato juice into wheat flour, respectively.

**TABLE 5:** Effect of different potato flour/wheat flour ratios on noodles' color.

Treatment	L*	a*	b*
Control (100% wheat)	67.77 ± 0.23^a^	3.64 ± 0.01^f^	27.67 ± 0.72^d^
Wheat 90%_FD_Potato 10%	61.57 ± 0.93^b^	5.36 ± 0.04^d^	28.21 ± 0.18^cd^
Wheat 70%_FD_Potato 30%	58.14 ± 0.25^d^	7.49 ± 0.06^b^	31.46 ± 0.06^a^
Wheat 50%_FD_Potato 50%	57.31 ± 0.11^e^	6.44 ± 0.03^c^	28.21 ± 0.02^cd^
Wheat 90%_LTB_Potato 10%	60.29 ± 0.01^c^	4.44 ± 0.01^e^	26.77 ± 0.03^e^
Wheat 70%_LTB_Potato 30%	57.56 ± 0.01^de^	6.59 ± 0.01^c^	28.86 ± 0.01^bc^
Wheat 50%_LTB_Potato 50%	56.49 ± 0.01^f^	8.02 ± 0.01^a^	29.35 ± 0.02^b^

*Note*: The results are the means of three determinations ± standard error. A mean value with a different letter in the same column indicates a significant difference (*p* < .05, *n* = 3).

Abbreviations: FD, Freeze‐dried; LTB_OD, Low‐temperature blanching then oven drying.

A significant increase in the amount of potato flour caused a significant increase in the redness (a*) and yellowness (b*) values. The color of the noodles gradually became more yellow. The yellowness was more pronounced for noodles substituted with LTB_OD potato flour, mostly because this flour was yellow compared to freeze‐dried potato flour. Collins and Pangloli (1997) mentioned that increasing yellowness in noodles could be due to the naturally occurring components in sweet potato flour. Pangloli et al. (2000) reported that yellow‐colored noodles may result from interactions between non‐wheat and wheat components, such as polyphenol oxidase (PPO). During the noodles' frying process, the sugars in potato flour will likely combine with the protein in the system to produce the Maillard reaction (Kolarič et al., 2020), producing a yellow color. Additionally, the yellow color of potato flour may explain the difference in redness and yellowness in cooked noodles (Nawaz et al., 2019).

#### 3.5.2 Cooking properties

The results of the cooking properties of noodles are presented in Table 6. It was found that the water absorption of noodles was greater when wheat flour was substituted with potato flour. When noodles are cooked, starch gelatinizes and gluten swells (Pu et al., 2017). Noodles with 30% freeze‐dried potato flour had the highest weight (93.83% ± 0.51), while the control had the lowest weight (85.81 ± 0.54). Adding potato flour increases the noodles' ability to bind water during cooking (Pu et al., 2017). The higher cooking weight of the potato blend noodles could be attributed to the potato starch's higher swelling and viscosity profiles (Sandhu et al., 2010). However, substituting freeze‐dried potato flour beyond 30% slightly reduced the cooking weight of the potato blend noodles from 93.83% ± 0.51 to 92.99% ± 0.37. The reason could be the decreased gluten protein content that weakens the gluten network (Majzoobi et al., 2011).

**TABLE 6:** Water absorption capacity and cooking loss of noodles made from wheat/potato flour blends.

Samples	Water absorption capacity (%)	Cooking loss (%)
Control (100% wheat)	85.81 ± 0.54^b^	2.61 ± 0.17^e^
Wheat 90%_FD_Potato 10%	91.81 ± 0.52^a^	3.76 ± 0.08^d^
Wheat 70%_FD_Potato 30%	93.83 ± 0.51^a^	5.67 ± 0.16^c^
Wheat 50%_FD_Potato 50%	92.99 ± 0.37^a^	6.64 ± 0.18^b^
Wheat 90%_LTB_Potato 10%	88.74 ± 0.82^b^	3.85 ± 0.07^d^
Wheat 70%_LTB_Potato 30%	92.25 ± 0.97^a^	6.24 ± 0.12^bc^
Wheat 50%_LTB_Potato 50%	92.32 ± 0.23^a^	10.62 ± 0.09^a^

*Note*: The results are the means of three determinations ± standard error. A mean value with a different letter in the same column indicates a significant difference (*p* < .05, *n* = 3).

Abbreviations: FD, Freeze‐dried; LTB_OD, Low‐temperature blanching then oven drying.

Cooking loss refers to the amount of solid substances lost during the cooking process (Rombouts et al., 2014). Good‐quality noodles should lose a few solids in the cooking water. Noodle cooking loss significantly (*p* < .05) increased with potato flour replacement, as shown in Table 6. Noodles made with 50% of LTB_OD flour had the highest (10.62% ± 0.09) cooking loss, while the control had the least (2.61% ± 0.17). Noodles with high cooking loss will have a sticky texture due to their high solubility and low cooking tolerance (Sandhu et al., 2010).

The noodles made from LTB_OD potato flour had higher cooking loss than freeze‐dried potato flour. This could be due to the larger particle size of LTB_OD potato flour. Buzera et al. (2022) reported that bigger particles of potato flour dissolved more easily in water. In addition, Sandhu et al. (2010) reported that increasing potato starch decreased the noodles' cohesiveness. As a result, the noodles become loose and lose their microstructure, releasing more starch into the cooking liquid and increasing cooking loss.

### 3.6 Texture profile analysis

**FIGURE 6:**
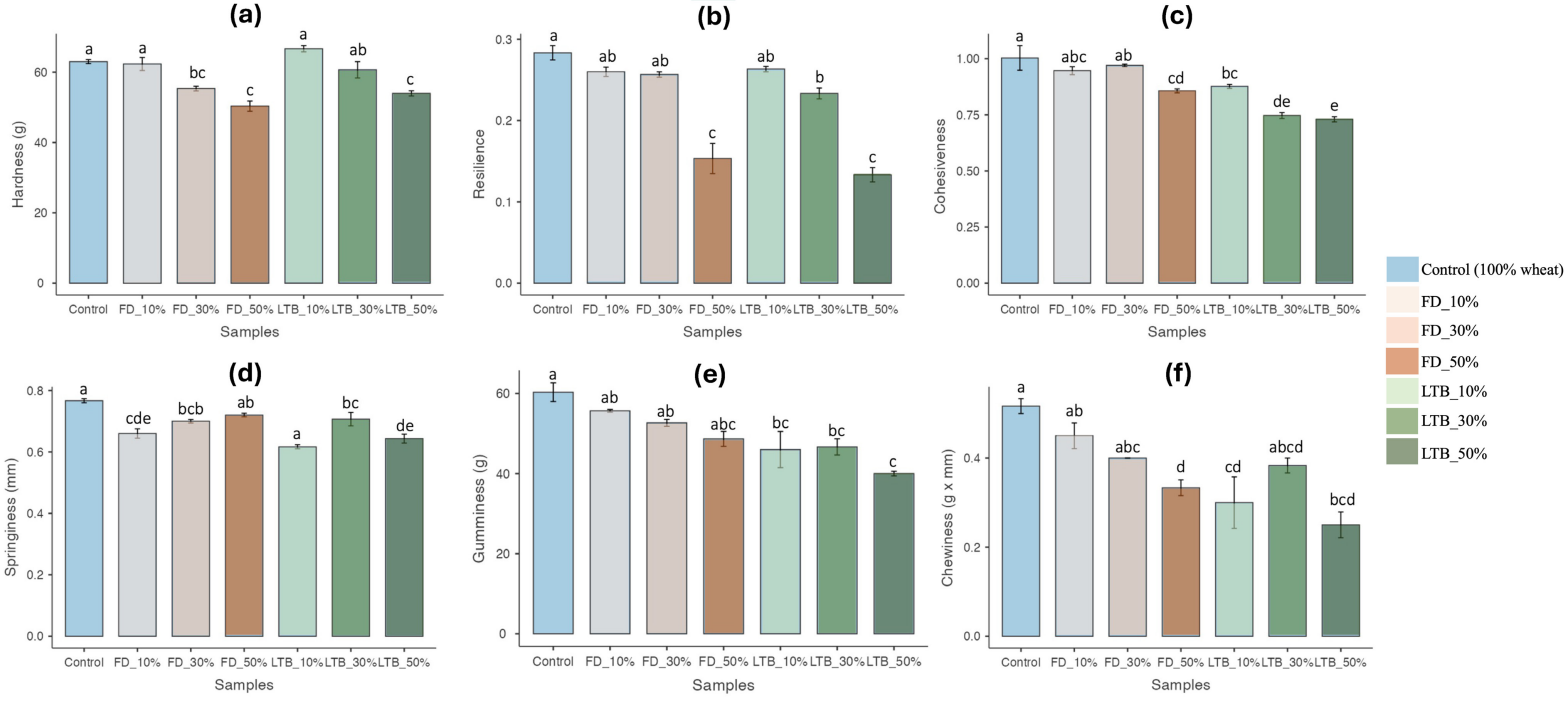
Texture profile analysis (TPA) parameters of cooked noodles with different potato flour/wheat flour ratios. (a: Hardness, b: Resilience, c: Cohesiveness, d: Springiness, e: Gumminess, and f: Chewiness). The different letters indicate the siginificant difference.

Textural properties are considered one of the most critical characteristics in evaluating the quality and determining consumer acceptability of noodle products (Jia et al., 2019). Figure 6 shows the texture profile analysis (TPA) parameters of noodles made from the different wheat and potato flour blends. The value of all TPA parameters was less than those of control noodles, which were made of 100% commercial wheat flour. These results suggest that the noodles' textural attributes were affected by adding potato flour.

Noodles with 10% LTB_OD potato flour had the highest hardness values but did not significantly differ from the control and 10% freeze‐dried potato flour noodles (Figure 6a). Noodles with 50% freeze‐dried and LTB_OD potato flour had lower hardness and significantly differed from the other flour blends. Hardness corresponds to the stress required to deform a food to a certain deformation (Funami & Nakauma, 2022). Hardness is considered a key indicator of the overall texture quality of noodles (Zou et al., 2021). Similar results were reported by Nawaz et al. (2019), who attributed the decrease in hardness to the weakening of the gluten network and disulfide bonding in the noodles. Deng et al. (2023) reported that when modified potato flour is added in excess, wheat flour loses its gluten protein content, and the dough network weakens, resulting in noodles with poor texture characteristics. On the other hand, an increase in hardness with the substitution of wheat flour with potato flour has been reported by Pu et al. (2017). Deng et al. (2023) mentioned that by interacting with potato and wheat flour and increasing water absorption in the potato–wheat system, starch particles would fill in the gluten network, reducing dough elasticity and hindering the formation of gluten networks, which results in a reduction in pores and an increase in noodles' hardness.

Resilience (Figure 6b) describes the rubbery state of the noodles and is a measure of recoverable energy after compression, providing insight into the structural matrix (Jia et al., 2019). In other terms, it explains how well a food product returns to its original shape after deformation. The resilience of noodles made with the different blends decreased with the addition of potato flour. However, replacing up to 30% of freeze‐dried potato flour gave noodles resilience comparable to the control noodles. Practically, the decrease in resilience in blended noodles means they will feel less springy or elastic when you bite into or manipulate them. They may deform more easily and not offer the same level of resistance to chewing as noodles made solely from 100% wheat flour. These effects may be positive for consumers who prefer a softer noodle texture (Koh et al., 2022).

Cohesiveness (Figure 6c) is defined as the strength of the internal bonds making up the body of the product (Funami & Nakauma, 2022). It measures how the noodle structure is disrupted in the first bite. The control noodles showed the highest cohesiveness and were not significantly different from noodles made from 10% and 30% freeze‐dried potato flour. Noodles made from LTB_OD flour blend had lower cohesiveness and decreased significantly compared to the control. This means these noodles are more prone to falling apart or disintegrating during handling or chewing. They tend to break apart more easily when you try to pick them up. The strength of the noodles depends on the starch–gluten network. Adding potato flour increases the starch content and dilutes the gluten content, weakening the starch–gluten network (Nawaz et al., 2019).

Springiness measures the ability of noodles to return to their original shape after compression. Springiness (Figure 6d) decreased with potato flour, but no significant changes were observed among the blend noodles. These results might be explained by the fact that potato flour reduces the gluten content in the dough, leading to a weaker network structure (Park et al., 2015).

The gumminess (Figure 6e) refers to the degree of stickiness. The gumminess of noodles made with the different blends decreases with the addition of potato flour. Noodles with less gumminess may feel smoother and less sticky in the mouth. This can be perceived as easier to chew and swallow as they don't adhere strongly to the teeth. Noodle chewiness (Figure 6f) indicates how much energy is needed to make them swallowable. It is associated with difficulty chewing the product (Joshi et al., 2018). The chewiness and gumminess values of all blend noodles were lower than those of the control noodles. Baik and Lee (2003) observed a linear relationship between chewiness and protein content. Gluten is responsible for dough elasticity and strength (Kovacs et al., 2004). Blending wheat flour with potato flour impairs the gluten matrix, weakening the noodle's texture. Xu et al. (2017) reported that other components in potato flour, such as fibers, protein, and gelatinized starch, could dilute the gluten content in dough samples, resulting in fragile noodles during cooking. Similarly, a study on the effect of different proportions of potato flour on the noodle's quality showed that the noodles deteriorated in texture when potato flour was more than 40% (Nawaz et al., 2019). Li et al. (2018) suggested adding potato flour spoiled noodles' textural attributes. They concluded that a high degree of gelatinization of potato flour could also weaken the gelling properties of noodles during cooking. Blending wheat flour with up to 30% freeze‐dried flour and 10% LTB_OD gave noodles that were better textured than the control.

### 3.7 Effect of flour particle size and pasting properties on noodles' quality

Based on the findings reported in Section 3.1, Hatcher et al. (2002) mentioned that particle size distribution influenced the textural properties of cooked noodles. In their study, noodles made from fine flour were significantly firmer and had higher compression resistance than those made from course flour (Hatcher et al., 2002). The particle size of flour also influences the rheology of dough (Cristiano et al., 2019). Chen et al. (2003) found that noodles made from small‐sized granule fractions had better noodle quality and fluidity than those made from large‐sized particles, which is attributed to larger specific surface areas of granules. Therefore, based on their particle size, freeze‐dried flours will likely produce noodles of better quality than LTB_OD flours.

In predicting noodle quality using pasting properties, Konik et al. (1992) showed that breakdown and final viscosity could be used to assess the quality of white salted Japanese noodles. In another study, Ross et al. (1997) observed a significant correlation between all pasting properties of flour and the surface smoothness, firmness, and elasticity of alkaline noodles. The best parameters for predicting alkaline noodles were breakdown and final viscosity. According to Zaidul et al. (2007), highly swollen starches make noodles smoother while reducing their firmness and elasticity, lowering their overall textural quality. This means flours with high viscosities should not be used for noodle making. Therefore, for noodles to be stable, a starch base must have limited swelling and viscosity that remain constant or even increase during continuous heating and shearing (Sandhu & Kaur, 2010).

### 3.8 Sensory analysis of cooked noodles

**FIGURE 7:**
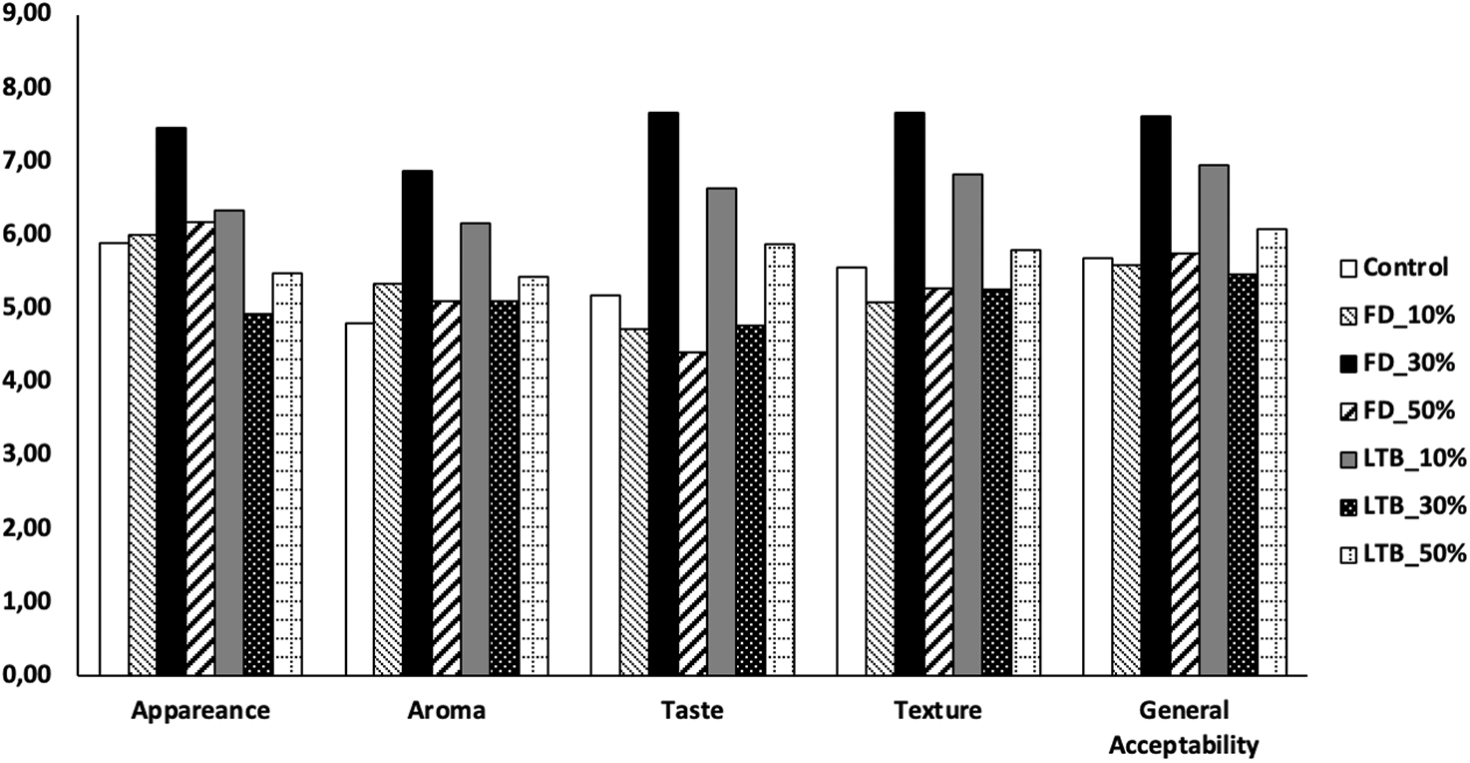
Sensory evaluation of cooked noodles.

The sensory characteristics of cooked noodles made from a mixture of wheat flour and potato flour are shown in Figure 7. The sensory assessment is a subjective estimation and assesses the overall acceptability of a food product. Therefore, it plays a significant role in selecting a food item (Sharif et al., 2017). Adding potato flour to wheat flour significantly improved the overall sensory scores of noodles. Noodles substituted with freeze‐dried potato flour up to 30% were the most accepted, followed by noodles substituted with 10% of LTB_OD. No significant difference (*p* ≤ .05) was reported between the control (100% wheat) and freeze‐dried potato flour at 10%. The aroma and texture of the noodles were the most affected sensory attributes when potato flour portions were increased. Substituting the noodles with 50% LTB_OD potato flour scored the lowest values in appearance and taste. Zhang et al. (2010) reported that consumers prefer bright, clear noodles without darkening or discoloration.

The texture of noodles substituted with freeze‐dried potato flour at 30% and LTB_10% was the most preferred, while the texture of noodles substituted with LTB_OD potato flour at 30% and 50% was the least preferred. The texture in sensory evaluation is related to the noodles' firmness (hardness). Khouryieh et al. (2006) reported that sensory scores, especially the texture of noodles, were comparable to instrumental textural scores (hardness in TPA profile). Texture profile analysis of noodles evaluates how firm or soft the noodles are, which differs from consumer to consumer. Some consumers prefer firm noodles, while others prefer soft ones (Wieser et al., 2020). Wootton and Wills (1999) found that Koreans prefer smooth‐textured noodles.

## 4 CONCLUSION

This study evaluated the effect of substituting wheat flour with potato flour processed differently. The LTB_OD flour had a larger particle size, increasing the mean particle size of the wheat–potato flour blends compared to freeze‐dried flour. Due to the high swelling and viscosity of potato starch, potato flour increased the pasting properties of flour blends. The brightness (L*) decreases while yellowness (b*) increases, especially in the noodles made from LTB_OD flour blends. Cooking loss and weight of cooked noodles increased, which can be attributed to the dilution of gluten and increased starch content. Noodle textural quality, such as hardness, cohesiveness, gumminess, and chewiness, decreased when the potato flour proportion reached 30%. Noodles made from 30% freeze‐dried flour gave the highest overall liking scores. Potato flour processed in different ways produced noodles of different qualities. Acceptable noodles were produced with 30% substitution with freeze‐dried flour and 10% replacement with low‐temperature blanched oven‐dried potato flour. Additional research to assess the nutritional profile and health benefits of the manufactured noodles is required.

## AUTHOR CONTRIBUTIONS

**Ariel Buzera:** Conceptualization (equal); data curation (equal); formal analysis (equal); investigation (equal); methodology (equal); software (equal); visualization (equal); writing – original draft (equal); writing – review and editing (equal). **Evelyne Gikundi:** Writing – review and editing (supporting). **Napoleon Kajunju:** Writing – review and editing (supporting). **Jackson Ishara:** Writing – review and editing (supporting). **Irene Orina:** Conceptualization (equal); methodology (equal); supervision (equal); writing – review and editing (equal). **Daniel Sila:** Conceptualization (equal); funding acquisition (lead); project administration (lead); resources (lead); supervision (lead); writing – review and editing (equal).

## FUNDING INFORMATION

This study was supported by the Japan International Cooperation Agency (JICA) (AFRICA‐ai‐JAPAN Project‐Phase 2).

## CONFLICT OF INTEREST STATEMENT

The authors declare that they do not have any conflicts of interest.

## Data Availability

Data are available from the corresponding author on request.
